# Traumatic Dislocation of the Hip in a Child Caused by Trivial Force for Age

**DOI:** 10.1155/2014/467246

**Published:** 2014-11-26

**Authors:** Hiroyuki Furuya, Yoshio Shimamura, Kazuo Kaneko, Hiroshi Sakuramoto, Kazuhiro Hirata, Yasuhisa Arai

**Affiliations:** ^1^Department of Orthopaedic Surgery, Tobu Chiiki Hospital, 5-14-1 Kameari, Katsushika-ku, Tokyo 125-8512, Japan; ^2^Department of Orthopaedic Surgery, Juntendo University School of Medicine, 2-1-1 Hongo, Bunkyo-ku, Tokyo 113-8421, Japan

## Abstract

Traumatic hip dislocation in children has a relatively rare occurrence. There are some residual complications, such as avascular necrosis of the femoral head, growth disturbance caused by premature fusion, neurological injury, recurrent dislocation, and posttraumatic arthritis. There is no consensus in the literature about the period of non-weight bearing after reduction. A rare case of a 13-year-old boy of hip dislocation caused by trivial force for age is reported followed by review of the pediatric literatures with treatment recommendation.

## 1. Introduction

The incidence of traumatic dislocation of the hip in children has low frequency compared to adults, less than 5% of all those dislocations [1, 2]. Lots of case reports were found; however, strategy of treatment has not been established [1–3]. We experienced a case of traumatic dislocation of the hip in a teenager. The case is reported followed by review of the literature with treatment recommendation.

## 2. Case Report

**Figure 1:**
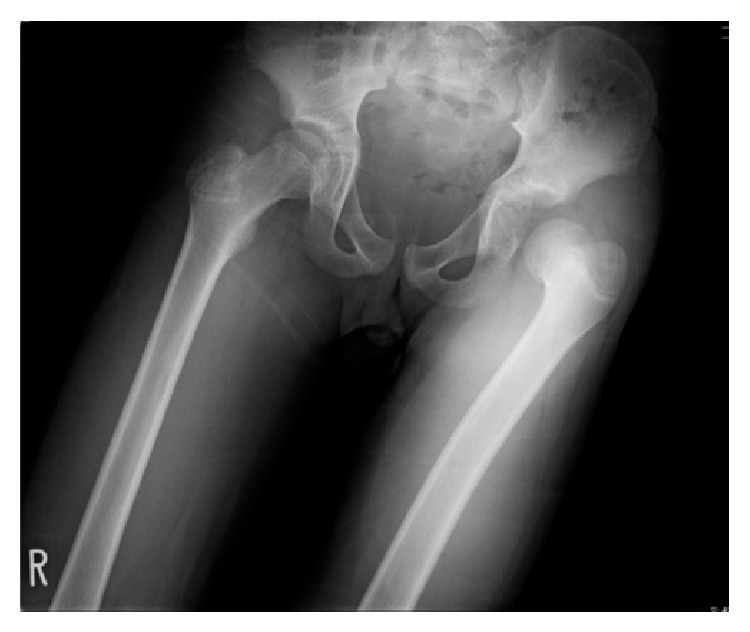
X-ray shows that the left femoral head is laterally displaced to the acetabulum, which is consistent with posterior dislocation of the hip.

**Figure 2:**
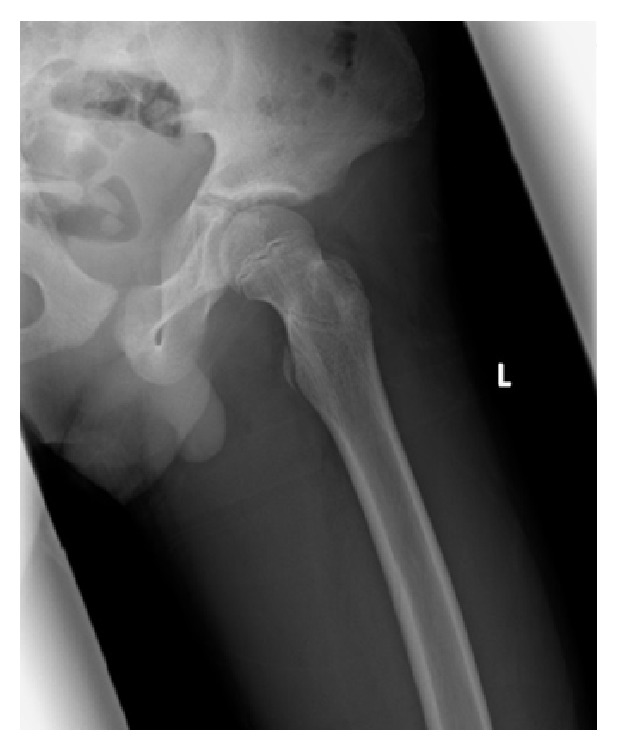
X-ray shows the affected side hip after reduction. The left femoral head is correctly positioned in the acetabulum.

**Figure 3:**
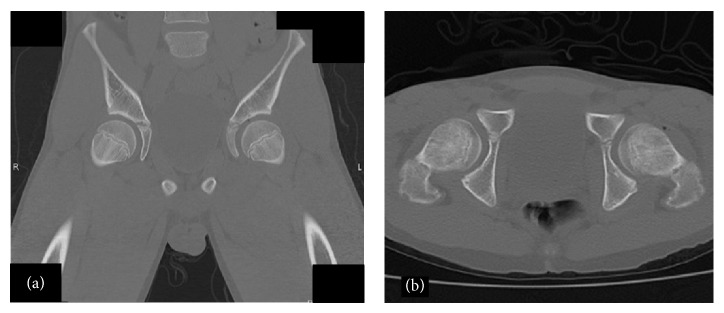
Coronal (a) and axial (b) CT scan after reduction shows the left femoral head correctly positioned in the acetabulum with no fractures or interposition of osteochondral fragments.

**Figure 4:**
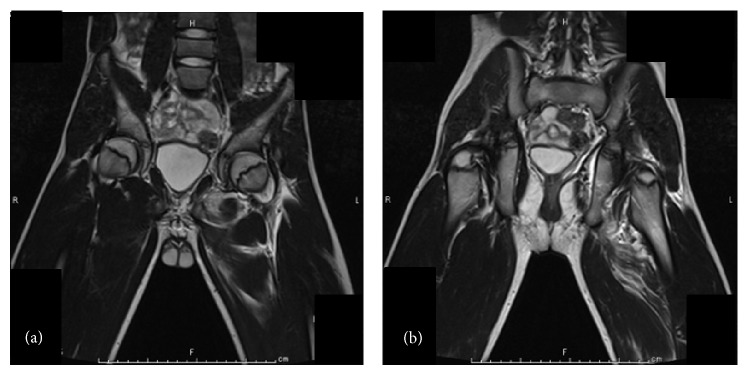
Coronal T2 weighted image in MRI shows no evidence of avascular necrosis of the femoral head (a) and damage of soft tissue (b) at 3 days.

**Figure 5:**
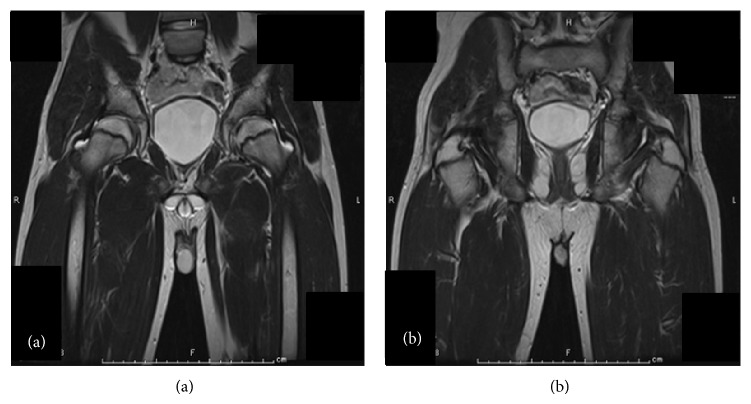
Coronal T2 weighted image in MRI shows no evidence of avascular necrosis of the femoral head (a) and healing of soft tissue damage (b) at 6 weeks.

A 13-year-old boy had slipped in gymnasium. He had pain in his left hip and was unable to walk. He was brought to the emergency department at an hour after injury with flexion, adduction, and internal rotation in his left hip. X-ray demonstrated that the left femoral head was laterally displaced to the acetabulum, which was consistent with posterior dislocation of the hip (Figure 1). Under general anesthesia and fluoroscopic guidance, the hip joint was gently reduced by Allis procedure at 2 hours after injury (Figure 2). After reduction of the joint, computed tomography (CT) was performed to evaluate the associated lesion (Figure 3). There were no fractures and no interposition space of osteochondral fragments. Magnetic resonance imaging (MRI) was demonstrated with no evidence of avascular necrosis of the femoral head and damage of soft tissue at 3 days (Figures 4(a) and 4(b)). Skin traction was taken for 3 weeks. And MRI demonstrated no evidence of avascular necrosis of the femoral head and healing of soft tissue damage at 6 weeks (Figures 5(a) and 5(b)). Then he was permitted partial weight bearing, resumed full weight bearing at 10 weeks, and was permitted sports activity at 12 weeks. He had no symptoms at his leg and hip joint and X-rays showed no significant change at 3 months.

The patient and the patients' parents were asked if data concerning the case could be submitted for publication and they consented.

## 3. Discussion

The dislocated hip accounts for 2–5% of all joint dislocations, with only 5% of all traumatic hip dislocation occurring in children younger than 14 years [2]. In children, particularly those younger than 6 years, a trivial force combined with a minor mechanism (i.e., slipping on a wet floor) has been associated with traumatic hip dislocation, and the greater the force required in older child. Athletic injuries and falls from significant heights are the most common cause in 6–10-year-old children, and injuries related to motor vehicle accident are common in children older than 10 years [2]. Moseley [5] categorized the causes of traumatic hip dislocation in children into falls (50%), motor vehicle accidents (30%), and sports and recreation (18%).

Diagnosis of traumatic hip dislocation with or without fracture is confirmed by X-ray. Superior lateral displacement of the femoral head with respect to the acetabulum is consistent with posterior dislocation. CT or MRI provides better delineation of fragmented osseocartilaginous structures that may not be apparent on X-ray and has been recommended after closed reduction.  Table 1 lists the different types of posterior hip dislocations [4, 6].

As for treatment, traumatic dislocation of the hip in children is recommended early closed reduction under intravenous sedation or general anesthesia. When the dislocation cannot be reduced by closed reduction, open reduction should be converted. Postreduction X-ray, CT, and MRI should be done for assessment of the complication of the joint, the presence of bone fragments, and damage of soft tissues.

With regard to rehabilitation, no literature suggested when weight bearing will be permitted. Some authors suggested a period of up to 4 months, and others suggested that the non-weight bearing period is unrelated to the outcome [3, 7]. Glass suggested that no weight bearing period was need 4 to 6 weeks for the soft tissue injury to heal [8]. One regime involves 5 weeks of bed rest with or without traction followed by a few weeks of partial weight bearing allowing the synovial irritation and soft tissue to heal [7]. We believe that rest and immobilization by skin traction for a few weeks and non-weight bearing period for 6 weeks after reduction should be adopted as treatment of hip dislocation.

The most common complications after traumatic hip dislocation include avascular necrosis, growth disturbance with premature fusion, neurological injury, recurrent dislocation, and posttraumatic arthritis. The incidence of avascular necrosis after traumatic hip dislocation ranged from 5% to 10% of occurrence [3, 8]. The factors influence the severity of injury, the long time interval between injury and reduction, reduction method, and so on [9]. Barquet reported that avascular necrosis occurs in 6% of type I traumatic hip dislocation in children if reduction is successfully done within 4 hours, in 13% if reduction occurs 5–24 hours after injury, and in 66% if reduction is delayed for more than 24 hours [10]. Some studies have suggested that reduction procedure within 6 hours provides better results [6, 11]. As for the incidence of avascular necrosis, Stewart and Milford suggested that it was found to be 15.5% by closed methods and 40% by open reduction surgical procedure [4].

The incidence of sciatic nerve injury in the setting of posterior dislocation has been reported in up to 20% of the pediatric population and 13% of the adult population [3, 4, 10]. It is commonly neuropraxia and symptoms are transient.

There is little literature with sufficient followup about childhood traumatic dislocations. In some reports, cases of degenerative change are documented but usually in association with avascular change [3, 12].

In our case, the patient was treated based on the protocol of the scheme of Rieger et al. [2]. He had no symptoms and X-rays showed no significant change at present. Long-term followup is necessary by X-ray and termination of treatment is up to skeletal maturity.

## 4. Conclusion

Traumatic hip dislocation is relatively rare in children and may present with a seemingly trivial mechanism. Regardless of age, the clinical entity constitutes a true orthopedic emergency for which attempt closed reduction should be performed, ideally within 6 hours, under general anesthesia to minimize associated complications including avascular necrosis, posttraumatic arthritis, and long-term morbidity. Long-term followup is necessary by X-ray and termination of treatment is up to skeletal maturity.

## Figures and Tables

**Table 1:** Classification of traumatic dislocation of the hip [4].

Grade I	A dislocation with or without an insignificant chip fracture from the acetabular rim
Grade II	A dislocation with one or more large fragments from the acetabular rim, but with a sufficient socket remaining to ensure stability after reduction
Grade III	A dislocation with a blast fracture and disintegration of the acetabular rim that produces gross instability
Grade IV	A dislocation combined with a fracture of the neck or head of the femur
